# e-Beam and γ-rays Induced Synthesis and Catalytic Properties of Copper Nanoclusters-Deposited Composite Track-Etched Membranes

**DOI:** 10.3390/membranes13070659

**Published:** 2023-07-11

**Authors:** Nursanat Parmanbek, Nurgulim A. Aimanova, Anastassiya A. Mashentseva, Murat Barsbay, Fatima U. Abuova, Dinara T. Nurpeisova, Zhanar Ye. Jakupova, Maxim V. Zdorovets

**Affiliations:** 1 The Institute of Nuclear Physics of the Republic of Kazakhstan, Almaty 050032, Kazakhstanmzdorovets@inp.kz (M.V.Z.); 2 Department of Chemistry, L.N. Gumilyov Eurasian National University, Astana 010008, Kazakhstandjakupova_zh@enu.kz (Z.Y.J.); 3 Department of Nuclear Physics, New Materials and Technologies, L.N. Gumilyov Eurasian National University, Astana 010008, Kazakhstan; 4 Department of Chemistry, Hacettepe University, Ankara 06800, Turkey; mbarsbay@hacettepe.edu.tr; 5 Department of Intelligent Information Technologies, The Ural Federal University, 620002 Yekaterinburg, Russia; 6 Engineering Profile Laboratory, L.N. Gumilyov Eurasian National University, Astana 010008, Kazakhstan

**Keywords:** track-etched membranes, radiation-induced grafting, accelerated electrons, copper nanoclusters, methylene blue degradation

## Abstract

Effective removal of toxic inorganic and organic pollutants is one of the current leading challenges of wastewater treatment. In this study, the decomposition of methylene blue (MB) under UV light irradiation was investigated in the presence of copper nanoclusters (NCs)-deposited polyethylene terephthalate (PET) track-etched hybrid membranes. PET track-etched membranes (TeMs) with an average pore size of ~400 nm were grafted by functional acrylic acid (AA) monomer under electron beam irradiation after oxidation with H_2_O_2_/UV system. The radiation dose varied between 46 and 200 kGy. For the deposition of copper NCs, poly(acrylic acid) (PAA)-grafted membranes saturated with Cu(II) ions were irradiated either by electron beam or γ-rays to obtain copper-based NCs for the catalytic degradation of MB. Irradiation to 100 kGy with accelerated electrons resulted in the formation of small and uniform copper hydroxide (Cu(OH)_2_) nanoparticles homogeneously distributed over the entire volume of the template. On the other hand, irradiation under γ-rays yielded composites with copper NCs with a high degree of crystallinity. However, the size of the deposited NCs obtained by γ-irradiation was not uniform. Nanoparticles with the highest uniformity were obtained at 150 kGy dose. Detailed analysis by X-ray diffraction (XRD) and scanning electron microscopy (SEM) confirmed the loading of copper nanoparticles with an average size of 100 nm on the inner walls of nanochannels and on the surface of PET TeMs. Under UV light irradiation, composite membranes loaded with NCs exhibited high photocatalytic activity. It was determined that the highest catalytic activity was observed in the presence of Cu(OH)_2_@PET-*g*-PAA membrane obtained at 250 kGy. More than 91.9% of the initial dye was degraded when this hybrid membrane was employed for 180 min, while only 83.9% of MB was degraded under UV light using Cu@PET-*g*-PAA membrane. Cu(OH)_2_@PET-*g*-PAA membranes obtained under electron beam irradiation demonstrated a higher photocatalytic activity compared to Cu@PET-*g*-PAA membranes attained by γ-rays.

## 1. Introduction

Water treatment is the process of removing contaminants from water to make it safe for various uses, such as drinking, irrigation, and industrial processes. Synthetic dyes have been widely used in various industries, including textile, paper, and food, in recent decades [1]. Therefore, these dye molecules contribute significantly to water pollution due to their high reactivity and toxicity [2]. Many synthetic dyes are quite stable, so they remain in the environment for a long time and cause serious contamination [3]. Some synthetic dyes also exhibit carcinogenic and toxic properties that pose significant risks to human health due to functionalities such as aromatic compounds and benzidine [4]. Consequently, there is an urgent need to develop efficient methods to remove dye molecules from wastewater and prevent their harmful effects.

There are various approaches to water treatment, including physical, chemical, and biological methods [5,6]. Physical methods involve the removal of contaminants through physical barriers such as screens, filters, or membranes. Chemical methods include the use of chemical reactions, such as oxidation or reduction, to remove or convert contaminants [7]. Biological methods involve the use of microorganisms to degrade or remove pollutants [8,9]. In the case of synthetic dyes, chemical methods are often used to degrade them. One of the most effective and widely used chemical methods is the advanced oxidation process (AOP) [10,11,12], which involves the generation of highly reactive species, such as hydroxyl radicals, to degrade contaminants. AOP relies on combining hydrogen peroxide (H_2_O_2_) with ultra-violet light or ozone to remove hard-to-break pollutants from water. The effectiveness of AOP can be enhanced by using catalysts that improve the degradation rate of synthetic dyes. For example, catalysts based on metal–organic frameworks (MOFs) have been used effectively in this area recently [13]. MOFs are highly porous materials composed of metal ions and organic ligands, which can be designed to have certain advantageous properties such as high surface area, stability, and selectivity. MOFs have been shown to be effective catalysts for the degradation of various dyes such as triarylmethane and anthraquinone [6,14]. The catalytic activity of MOFs can be enhanced by doping with metal ions, such as Co, Cu, Ni, etc. Despite such promising materials and methods, current water treatment methods have worrisome shortcomings such as high operating costs, low efficacy, or potential to cause harmful accompanying pollutants to the environment; therefore, the development of new materials is still needed [15,16,17]. A promising and effective approach in water purification is the use of composite photocatalysts that can degrade different types of industrial dyes using light energy [18,19,20]. Photocatalysts based on composite membranes have been shown to be effective at decomposing various dye contaminants [21], including methylene blue (MB).

The development of highly effective and inexpensive new catalysts is crucial for the removal of synthetic dyes from water sources [22,23]. Porous membranes have unique properties that make them attractive for catalysis applications, particularly hybrid membranes decorated with metal nanoparticles. They have a large surface area and well-defined pore structures that can be tailored for specific applications. Pore size and distribution can be controlled to allow efficient diffusion of reactants and products across the membrane while providing a high surface area for catalytic reactions to occur. In addition, the porous nature of these membranes can provide a protective environment for the nanoparticles, prevent aggregation, and increase their stability and reusability [24,25,26]. This makes them particularly useful as support materials for heterogeneous catalysts immobilized on solid supports [27,28,29].

The use of metal nanoparticles in the manufacture of polymeric membranes is a growing area of research and development in membrane technology [30]. The addition of metal nanoparticles to polymeric membranes can improve their performance in several ways, including enhancing flux (permeation rate of the membrane) and reducing fouling (accumulation of unwanted materials on the membrane surface) [31,32]. Metal nanoparticles can be incorporated into polymeric membranes through various techniques, including blending, in situ synthesis, and coating [25,33,34]. The choice of technique depends on several factors, such as the properties of the nanoparticles, the polymeric membrane matrix, and the intended application. Their loading into polymeric membranes can impart unique properties such as antibacterial, antifouling, and catalytic activity to the membranes. For example, it has been shown that silver nanoparticles have antibacterial properties and can be used to prevent bacterial fouling of the membrane and to remove organic dyes such as methylene blue [21] and heavy ions such as arsenic from aqueous media [35].

The synthesis of stable nanoclusters of metals with controlled size, shape, and homogeneity remains a challenge, although significant progress has been made in recent years and a variety of promising approaches are being explored [36,37]. This is because the properties of metal nanoclusters can be highly dependent on their size and shape, and even small variations in synthesis conditions can lead to significant changes in these parameters [38]. In addition, metal nanoclusters are often prone to aggregation or coalescence, which can limit their stability and homogeneity. To overcome these challenges, researchers have developed several approaches to synthesize stable metal nanoclusters. One common method is to use protective ligands, such as thiol molecules, to stabilize the nanoclusters and prevent aggregation. Another approach is to use a matrix material, such as a zeolite or a polymer, to provide a stable environment for the nanoclusters [39]. Besides stabilizing the nanoclusters, controlling their size and shape is also important to achieve the desired properties. This can be accomplished by various methods, such as controlling reaction conditions, using templates or molds to define the shape, or using surfactants or other additives to control the growth of the nanoclusters. Among the many techniques proposed for the synthesis of NPs, radiation-induced synthesis occupies a special place [40,41]. Radiation–chemical reduction of many metal ions in aqueous solutions in the presence of stabilizers leads to the formation of metal sols [37]. This method of obtaining metallic NPs has a number of well-known advantages that provide a fairly wide range of application [42]. In general, the radiation–chemical synthesis of metal nanocrystals in templates includes several successive stages, such as fabrication of a template with a highly hydrophilic surface, sorption of metal ions from saturated solutions of precursors, and treatment of the reaction mixture with ionizing radiation (electrons, x-rays, and gamma radiation) [40]. Previously, the use of gamma radiation (Co^60^) to produce copper nanoclusters in PAA-grafted PET TeMs was demonstrated [24]. Therefore, in our current study, the irradiation process with accelerated electrons for the formation of copper-based NCs was investigated in detail, while previously optimized conditions were used for the gamma-induced synthesis of NCs.

The aim of this study is to evaluate the potential of hybrid composites based on polyethylene terephthalate (PET) track-etched membranes (TeMs) with deposited copper nanoclusters (NCs) as catalysts for the photodegradation of methylene blue (MB) under UV light. PET TeMs are a type of membrane created using heavy ion radiation and are known for their high chemical, UV, and thermal stability [43], making them useful in a variety of applications. The addition of copper nanoclusters to these membranes confers catalytic activity to them [44], potentially leading to improved performance in the decomposition of various classes of toxic pollutants [45,46]. The controlled pore size, high surface area, and physical integrity are the main advantages of TeMs. In order to obtain hybrid composite membranes that combine the advantages of TeMs and NCs, the membranes were first chemically functionalized by grafting with poly(acrylic acid) (PAA) to obtain a higher NCs loading and increase their stability. NPs can be stabilized with polymeric brushes that interact strongly with them, controlling their growth and preventing them from aggregating. The stabilizing polymer matrix plays a crucial role in the catalytic performance of NPs along with their size and shape [47]. Besides being a highly effective sorbent, PAA is also a well-known polymeric stabilizing matrix for nanoparticles [48]. Copper-based NCs were loaded onto PAA-grafted TeMs (PET-*g*-PAA) by electron beam irradiation or γ-rays treatment to provide a stable and promising catalyst for the degradation of MB. The results of this study provide important implications for obtaining new and improved catalysts for various chemical reactions.

## 2. Materials and Methods

### 2.1. Materials

Acrylic acid (AA), hydrogen peroxide (H_2_O_2_)_,_ copper sulfate (CuSO_4_·5H_2_O), methylene blue (MB), and all other chemicals were purchased from Sigma-Aldrich (Schnelldorf, Germany). AA was distilled under vacuum just before use. All aqueous solutions were prepared using deionized water (18.2 MΩ/cm, Aquilon D-301) obtained using “Aquilon—D301” water purification system with a resistivity of 18.2 MΩ/cm (Aquilon, Podolsk, Russia).

To obtain track-etched membranes, a PET Hostaphan^®^ RNK film («Mitsubishi Polyester Film», (Wiesbaden, Germany) was used as the polymer template. The film was irradiated with ^84^Kr^15+^ ions at a specific energy and fluency using a cyclotron (Cyclotron DC-60, Institute of Nuclear Physics of Kazakhstan). After irradiation, the film was etched in 2.2 M NaOH to create nanopores across the cross-section, resulting in PET track-etched membranes (TeMs) with an average pore diameter of 385 ± 9 nm.

### 2.2. Electron Beam-Induced Grafting of Acrylic Acid

For the modification of the surface and pore interior of PET templates with functional PAA polymer, simultaneous grafting method by direct irradiation with accelerated electrons was performed. Prior to irradiation, the surface of PET TeMs was oxidized with H_2_O_2_ according to the method described elsewhere [49], and all samples were thoroughly cleaned with acetone in an ultrasonic bath and immersed in an aqueous solution of AA at various concentrations (5–20, wt.%). In order to prevent the homopolymerization of AA, 1% (wt.%) CuSO_4_ was added to the reaction mixture. Then, the reaction mixture was purged with argon to remove dissolved oxygen. Samples were irradiated on an ILU-10 linear electron accelerator up to various doses.

After irradiation, the samples were kept in water for 24 h at 60 °C and dried under vacuum till a constant weight. The degree of grafting (*DG*) was determined gravimetrically according to the following Equation (1):

(1) $$DG=\frac{m-m_{0}}{m_{0}}\times 100\%$$

where $m_{0}$ and m are the masses of PET TeM before and after grafting, respectively.

The irradiation dose varied from 46 to 200 kGy. The samples were irradiated at the ILU-10 electron accelerator (Kurchatov, Kazakhstan), where the electron beam energy was 3.8 MeV and the average electron beam current was 6.84 mA. The irradiation dose was varied by changing the number of runs of the sample holder under the electron beam at a constant speed and controlled by B3WinDose electronic dosimeters (Gex, Palm City, FL, USA). In all experiments performed, dose measurement error did not exceed 10%. Since the maximum dose measured by this type of dosimeter is 150 kGy, a calibration curve was plotted to calculate doses in experiments above 150 kGy (Figure S1).

### 2.3. Radiation-Induced Synthesis of Copper Nanoclusters (NCs)

#### e-Beam and γ-rays Treatment of PET-g-PAA TeMs

PET-*g*-PAA TeMs obtained in Section 2.2 were used as templates for the formation of NCs. Sorption of Cu(II) ions was accomplished by passing a saturated solution of copper sulfate (37%, *w/v*) for at least 48 h through the pores of the PAA-grafted membranes using a LOIP peristaltic pump (JSC «Laboratory Equipment and Instruments»б Saint-Petersburg, Russia). The waiting time of the samples in saturated CuSO_4_ solution without using a pump was at least 15 days. Irradiation was carried out using ILU-10 accelerator (JSC “Park of Nuclear Technologies”, Kurchatov). Each membrane was immersed into glass vials containing 0.01 M CuSO_4_ in 10% EtOH, bubbled with argon, and irradiated by e-beam or γ-rays. For e-beam irradiation, beam current, conveyor speed, and electron energy was 6.84 mA, 7.0 m/min and 3.8 MeV, respectively. The samples were irradiated till different total absorbed doses of 100, 128.3, 185, and 231.2 kGy. γ-rays treatment was carried out in gamma mode using a tantalum bremsstrahlung converter manufactured by the INP named after Budker SB RAS at the ILU-10 accelerator. During irradiation with gamma quanta, a dose range of 100–200 kGy was employed, since destruction of the PET polymer template occurred at radiation doses exceeding 200 kGy.

The amount of copper deposited was determined gravimetrically with an accuracy of 0.1 mg (AS 220.R2, Radwag, Radom, Poland) and expressed in mg/cm^2^ based on the difference in the weights of the PAA-grafted membranes before and after e-beam and γ-rays treatments.

### 2.4. Characterization

The morphology of the grafted and copper NCs-loaded hybrid membranes were investigated by JEOL JSM-7500F scanning electron microscopy (SEM) (Tokyo, Japan). SEM was used for visualization of distribution of copper NCs along the nanochannels of PET-*g*-PAA TeMs.

The crystal structure of the NCs was examined on a D8 Advance diffractometer (Bruker, Karlsruhe, Germany) in the angular range of 2θ 30–80° with a step of 2θ = 0.02° (measuring time: 1 s, tube mode: 40 kV, 40 mA). The mean size of crystallites was determined via the broadening of X-ray diffraction reflections using the Scherer equation. The phase composition was determined using the Rietveld method, which is based on approximating the areas of the diffraction peaks and determining the convergence with reference values for each phase. The volume fraction of the composite phase was determined using the following Equation (2):

(2) $$V_{admixture}=\frac{{RI}_{phase}}{I_{admixture}+{RI}_{phase}}$$

where $I_{phase}$ is the average integral intensity of the main phase of the diffraction line, $I_{admixture\;}$is the average integral intensity of the additional phase, and *R* is the structural coefficient equal to 2.

Tensile strength measurements were carried out using a special tensile testing machine presented in Figure S2. It directly gave the value of breaking force for the standard circle samples of the investigated PAA-grafted membranes. The results of three measurements were averaged and given in units of pressure.

### 2.5. Photocatalytic Activity

Methylene blue (MB) was used as a model dye to examine the photocatalytic activity of as-prepared composite membranes under UV light irradiation. All experiments were carried out in 200 mL double-walled glassware. A 300 W high-pressure UV lamp (Ultra-Vitalux 300 W, Osram, Augsburg, Germany) was used as the source of UV light. The distance from the light source to the solution was 15 cm. In a typical procedure, a 1 × 1 cm composite membrane was immersed in 20.0 mL of MB solution at a concentration of 3.0 mg/L and stirred for 30 min in the dark to achieve adsorption–desorption equilibrium between the organic dye and the catalyst (a laboratory setup is presented on the Figure S3). After irradiation with UV light, a 0.35 mL aliquot was taken from the reaction mixture every 5–10 min and its absorbance was measured using a Specord-250 spectrophotometer (Jena Analytic, Jena, Germany) in the wavelength range of 200–800 nm. According to the Beer–Lambert law, the concentration of MB is directly proportional to its absorbance; thus, the degree of degradation (D, %) of MB was calculated relative to its characteristic peak at 664 nm using the following Equation (3) [21]:

(3) $$D=\frac{C_{0}-C}{C_{0}}\times 100\%=\frac{A_{0}-A}{A_{0}}\times 100\%,$$

where *A*_0_ is the initial absorbance of MB solution at 664 nm before loading the catalyst, *A* is the absorbance at 664 nm at different time intervals, and *C*_0_ is the concentration of feed solution.

## 3. Results and Discussion

### 3.1. Synthesis of PET-g-PAA TeMs by e-Beam-Mediated Grafting and Loading Them with NCs by e-Beam and γ-rays Treatments

**Figure 1:**
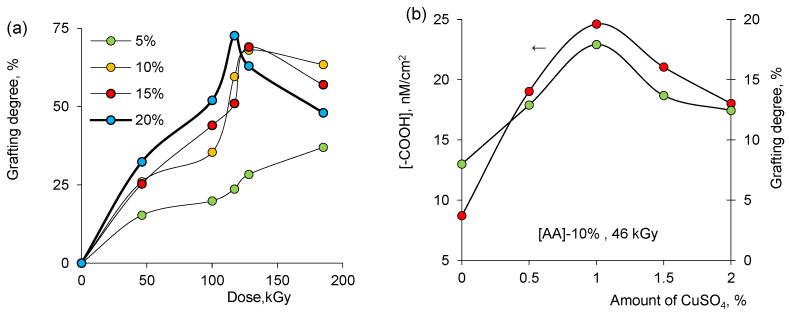
Effect of the irradiation dose on the degree of grafting to PET TeMs at various AA concentrations (**a**). Effect of concentration of the CuSO_4_ inhibitor on the concentration of terminal carboxyl groups and the degree of grafting (**b**).

Radiation-induced grafting is a widely used approach to impart desired surface functionalities to base polymers as it has many advantages over conventional chemical processes, such as environmental friendliness, operation at or below room temperature without any catalyst or initiator, low cost, simplicity, and ease of control of material composition and process [50,51]. The degree of grafting (DG) is an important expression that indicates the amount of new functionality imparted to the surface. It has been frequently reported that there is a competition between homopolymerization and graft copolymerization during the radiation-induced grafting process. The increase in homopolymerization results in a lower grafting, while the reverse case yields higher grafting rates. The variation of the degree of grafting (DG, %) is shown in Figure 1a as a function of absorbed dose under a beam of accelerated electrons. A downward trend in DG was observed in solutions with a high concentration of AA above a dose of about 128 kGy. This can be associated with the predominance of homopolymerization [52,53] and shows that increasing monomer concentration causes polymerization to progress against grafting at high radiation doses. This assumption was followed and confirmed by the reduction in the concentration of terminal carboxyl –COOH groups determined by the toluidine blue (TB) method [54]. We similarly validated the degrees of grafting obtained by quantifying the -COOH groups in PAA-grafted membranes, as will be discussed later Figure 1a also shows that DG increases with AA concentration up to about 128 kGy. In order to inhibit homopolymerization of AA, CuSO_4_ was added to the grafting solution. As can be seen from Figure 1b, DG is significantly affected by the concentration of the inhibitor. CuSO_4_ not only reduces the homopolymerization rate but also the rate of graft copolymerization. The difference between the inhibition rates of grafting and homopolymerization will determine the increase in weight of the polymer to be grafted to the substrate. From the results in Figure 1a, it is seen that the simultaneous decrease in homopolymerization and graft copolymerization rates up to a concentration of approximately 1% CuSO_4_ promotes grafting, an equilibrium is reached at this concentration, and then the addition of more inhibitors impedes grafting. Therefore, the optimum amount of CuSO_4_ was determined as 1%.

**Figure 2:**
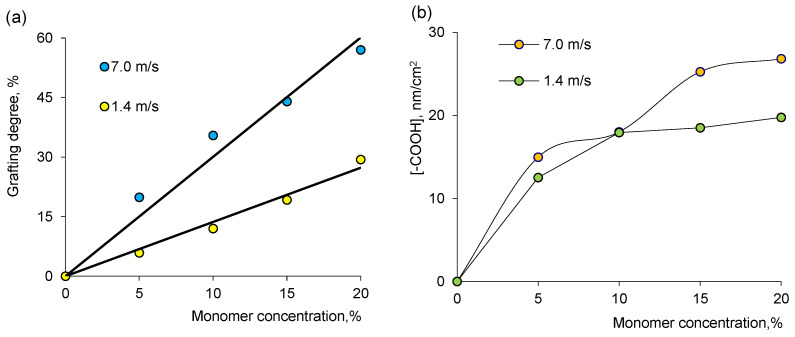
Effect of AA concentration on DG (**a**). The change of -COOH concentration in PAA-grafted PET TeM membranes as a function of AA concentration in grafting solution (**b**) at different speeds of the beam section of the conveyor of the ILU-10 accelerator.

An increase in AA concentration increases the degree of grafting as expected, as more monomers will be available for grafting in the immediate vicinity of the PET substrate (Figure 2a). As can be seen in Figure 2a,b, the effect of the beam section of the accelerator conveyor on the DG value was also examined. At a speed of 1.4 m/s for one pass of the conveyor section, the irradiation dose was about 46–50 kGy, whereas at a speed of 7.0 m/s for one “run” of the conveyor section under the electron beam, the irradiation dose of the sample did not exceed 4.8–5.0 kGy. Thus, at an accelerator conveyor speed of 7.0 m/s, the minimum dose of 46.2 kGy accumulated only after 10 runs of the sample under the beam. The data presented in Figure 2 indicated that in contrast to a single “run” at low speed, the degree of grafting of AA to PET increased significantly (about 70–80%) by repeatedly passing the samples under an accelerated electron beam at a high conveyor speed.

**Figure 3:**
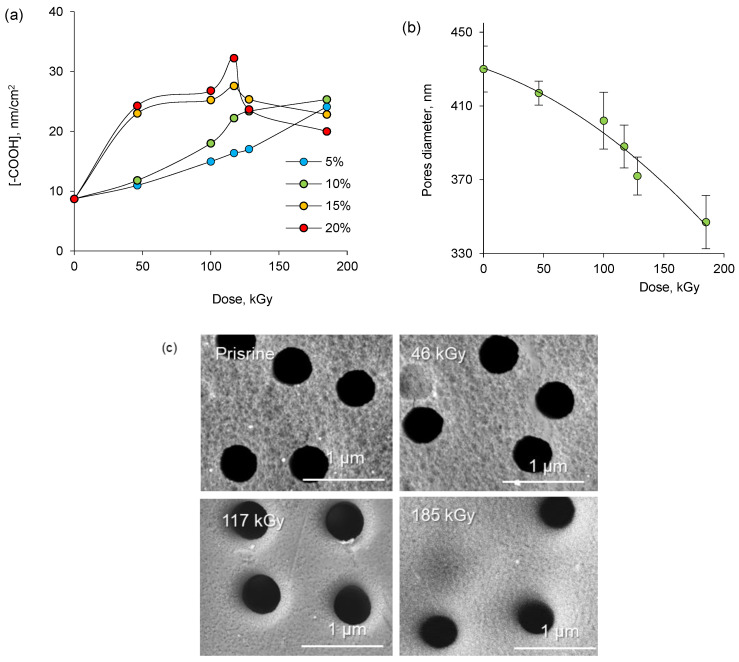
Change of -COOH concentration of PAA-grafted PET TeMs as a function of absorbed radiation dose (**a**); change of pore diameter of PAA-grafted PET membranes as a function of absorbed radiation dose (concentration of AA—10%; CuSO_4_—1%) (**b**); and SEM images of PAA-grafted PET TeMs obtained at the different doses (**c**).

Analysis of PAA-grafted membranes using the TB dye shows that the concentration of -COOH groups increases continuously only in solutions with low concentration of AA at irradiation doses up to 185 kGy, as can be seen in Figure 3a. However, -COOH concentration decreased after 128 kGy in PAA-grafted membranes prepared in solutions with high AA concentration (10–15%), which is in good agreement with the results presented in Figure 1a and is attributed to increased homopolymerization in solution and hence reduced grafting. One of the important parameters for evaluating the effectiveness of TeMs modification is the examination of the decrease in pore size due to grafting. The change in the pore diameter during grafting was assessed by scanning electron microscopy (SEM). Measurements were carried out from two different positions of the sample and five pores in each case (Figure 3c). The mean pore diameter of the pristine PET TeM was 430 nm. As a result of grafting, the pore diameter decreased to 417 nm at 46 kGy irradiation. Further irradiation caused the pore diameter to decrease to 388 nm at 117 kGy, and then to 335–347 at 185 kGy. The results indicate that the surface and pore walls of PET TeM are coated with PAA grafts depending on the irradiation dose, and it seems possible to control the thickness of the grafted PAA layer.

**Figure 4:**
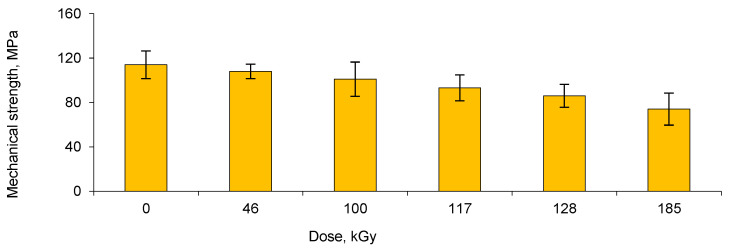
Change in tensile strength of membranes depending on the e-beam radiation dose applied during the synthesis of PAA-grafted PET TeMs (concentration of AA—10%).

The changes in the mechanical properties of the membranes were investigated by tensile strength measurements. As can be seen in Figure 4, the mechanical strength of the membranes gradually decreased with increasing e-beam irradiation dose. In addition, it is worth noting that grafted membranes obtained at a dose of 185 kGy become brittle. The mechanical distortions observed at high radiation doses limited the radiation dose used in further grafting experiments. Consequently, the optimal conditions for e-beam-induced grafting of PET TeMs were determined by considering both the degrees of grafting and the mechanical stability of the membranes. These conditions were determined as the inhibitor concentration of CuSO_4_ not being more than 1%, the monomer concentration of AA not exceeding 10%, and the radiation dose between 100–117 kGy, at which a sufficiently high degree of PAA grafting was achieved while maintaining the mechanical strength of the polymer template.

### 3.2. Deposition and Characterization of Copper NCs

**Figure 5:**
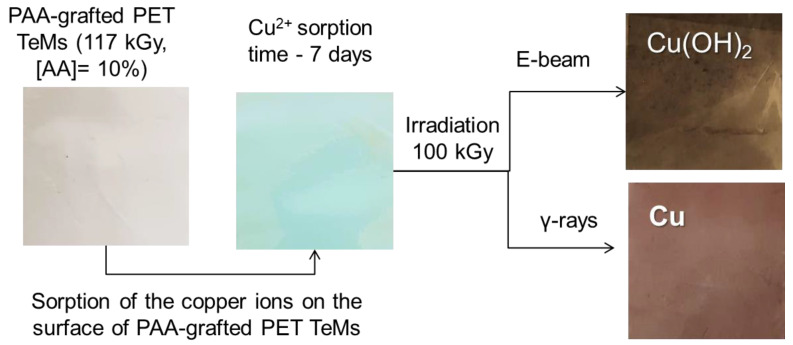
General production scheme of the composite hybrid membranes using e-beam and gamma-rays and the digital pictures of the films obtained during the production stages.

After obtaining PAA-grafted membranes by e-beam-induced grafting, the nanochannels and surface of these membranes were loaded with Cu NCs. Loading of copper NCs was accomplished via sorption of Cu(II) ions at the highest possible amount by the PAA-grafted TeMs, followed by irradiation of the membranes under e-beam or gamma rays. The increase in the irradiation dose caused noticeable differences in the membranes. The smooth surface of the initial sample acquired a wavy structure with increasing radiation dose. The sorption of Cu(II) and subsequent radiation-induced loading of copper-based NCs could also be followed by significant changes in the color of the samples. The initial cloudy transparent appearance of PAA-grafted PET TeMs turned into a characteristic pale greenish color after complexation between PAA and Cu(II). Following the radiation treatment, the formation of copper-based NCs could be clearly indicated by the emerging brownish color, as seen in Figure 5.

**Figure 6:**
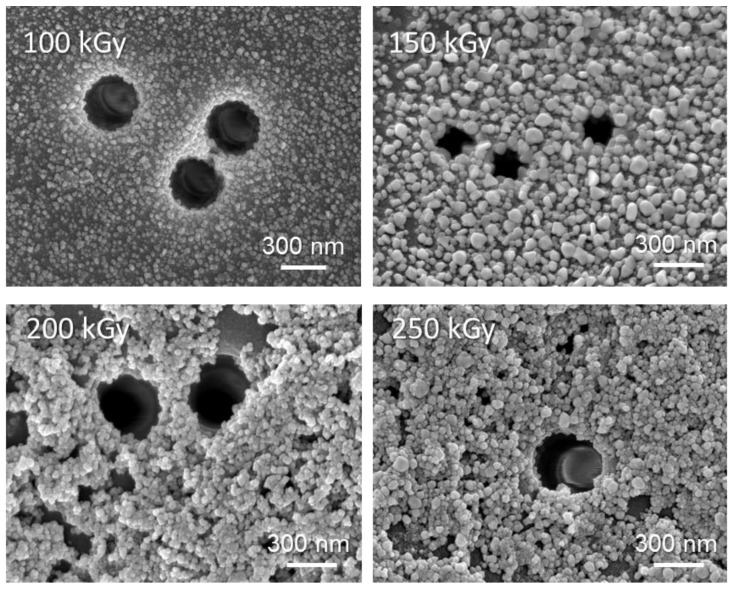
SEM images of Cu(OH)_2_@PET-*g*-PAA composite membranes obtained under e-beam irradiation.

The phase composition and crystal lattice parameters (crystallite size L, degree of crystallinity (CD), etc.) of the irradiated samples were studied by X-ray diffractometry and the results are presented in Table 1. All the diffraction peaks observed in e-beam-treated membranes could be indexed with orthorhombic Cu(OH)_2_ phase with symmetry group Cmcm(63) and cell constants (a) 2.950 Ǻ, (b) 10.439 Ǻ, and (c) 5.283 Ǻ, which are consistent with the standard card (JCPDS 35-0505). No other peaks were found in the diffraction pattern (Figure S4a). The blurring of the peaks indicated the formation of nanosized structures. Most likely, the appearance of the Cu(OH)_2_ phase occurred due to the instability of copper NPs. The change in the FWHM parameter of the main diffraction lines in the X-ray diffraction patterns indicated a change in the degree of crystallinity of the synthesized samples. By approximating the lines in the diffraction pattern with the required number of symmetric pseudo-Voigt functions, the width of the recorded FWHM lines was determined, which made it possible to characterize the perfection of the crystal structure and estimate the degree of crystallinity. As can be seen from the data presented in Table 1, the degree of crystallinity of Cu(OH)_2_ nanoclusters decreased with an increase in irradiation dose. Figure 6 shows SEM images of the surface of membranes with loaded Cu(OH)_2_ NPs. As can be seen from the presented images, the minimum sizes of NPs were observed at an irradiation dose of 100 kGy. The surface of the membrane and the nanochannel interiors were covered with a uniform layer of approximately 17 ± 3 nm nanoparticles, as can be seen in SEM images. At a dose of 200 kGy and higher, large aggregates of copper hydroxide were formed, and a significant portion of copper was reduced in solution rather than the membrane. SEM images of TeMs showed a substantial change in surface morphology and narrowing of the pores.

Irradiation with gamma quanta was performed in the dose range of 100–200 kGy, as destruction of the polymer template occurred at doses above 200 kGy. Data on the crystal structure of the resulting composite membranes (Table 2) revealed that copper nanoparticles and nanoclusters with a face-centered cubic (fcc) lattice and Fm-3m(225) symmetry group were formed upon irradiation with gamma-rays. The peaks (111), (200), (220), and (311) are the characteristic bands of the crystalline copper Cu^0^ phase (JPDF Cu № 04-0836). The typical XRD pattern of Cu@PET-*g*-PAA composite is shown in Figure S4b. The value of the unit cell parameter of the crystal lattice is also presented in Table 2. The deviation from the standard value of the lattice parameter at irradiation doses of 150–200 kGy is most likely due to uncompensated interatomic bonds of the surface atoms as well as the consequent reduction in distances between atomic planes near the surface of the particles.

**Figure 7:**
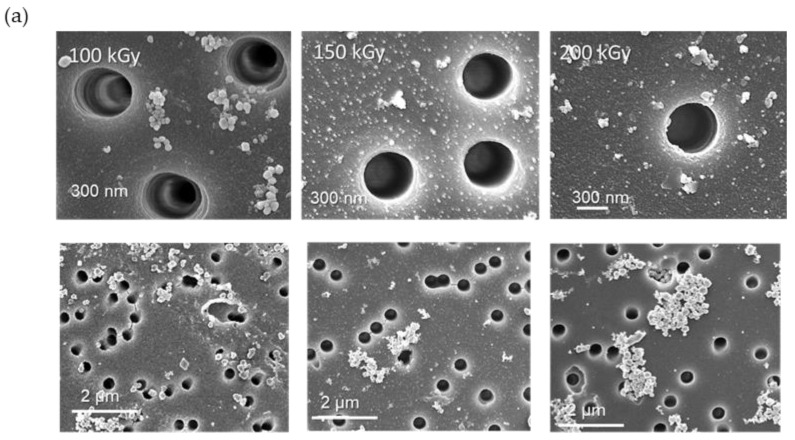

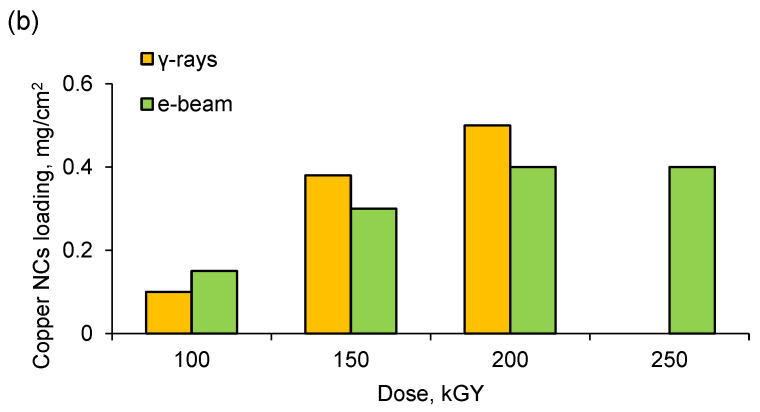
SEM images of Cu@PET-*g*-PAA composite membranes obtained under γ irradiation (**a**), Comparison of loading efficiencies of copper nanoclusters on PET-*g*-PAA membranes under gamma and e-beam irradiation (**b**).

Examination of the morphology of copper nanoclusters by SEM analysis revealed that the size of the nanoclusters obtained under γ-irradiation was not uniform and higher (~200–400 nm) compared to those obtained under e-beam, as can be seen in Figure 7a. Among the irradiation doses, 150 kGy seems to yield the most homogeneous nanoparticles. Figure 7b shows that in the case of irradiation by gamma rays, the reduction rate of copper is higher than in e-beam irradiation.

To summarize, according to the results of radiation-induced synthesis of copper NPs in nanochannels and on the surface of modified PET-*g*-PAA TeMs, it was observed that irradiation with accelerated electrons yielded uniform copper hydroxide nanoparticles homogeneously distributed over the entire surface of the template. The dimensions of Cu(OH)_2_ nanoparticles, which were approximately 17 ± 3 nm at 100 kGy dose, increased to about 100 nm at an irradiation dose above 200 kGy. On the other hand, irradiation with γ-rays yielded composites containing copper nanoclusters with a high degree of crystallinity (Figure 7). However, the size of the nanoclusters was not uniform. The most homogeneous nanoparticles were synthesized at 150 kGy.

### 3.3. Application of the Hybrid Composites for Methylene Blue Degradation

Industrial dyes are one of the dominant chemicals that make water unsuitable for humans and the environment. Among these dyes, MB is toxic, carcinogenic, and non-biodegradable, and above a certain concentration can cause destructive effects on human health and the ecosystem [55,56].

**Figure 8:**
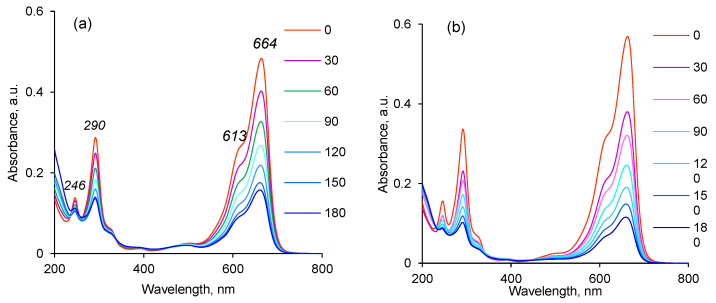

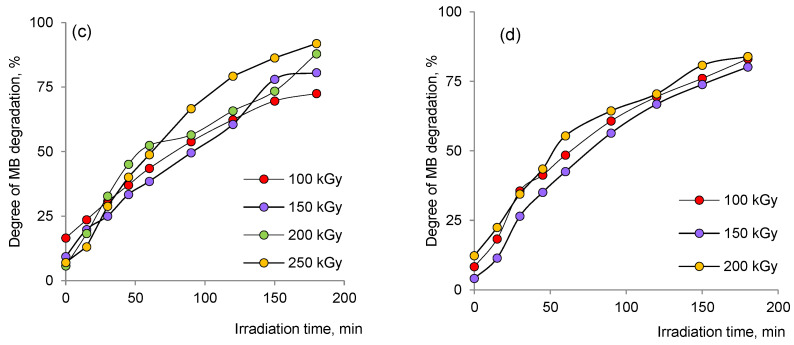
Typical absorption spectra for the decomposition of methylene blue (MB) in the presence of Cu(OH)_2_@PET-*g*-PAA (**a**) and Cu@PET-*g*-PAA (**b**) composite membrane (irradiation dose—100 kGy; temperature—30 °C; MB concentration—3 mg/L; size of the catalyst membrane—1 × 1 cm). The change of degree of dye decomposition (D, %) in the presence of Cu(OH)_2_@PET-*g*-PAA (**c**) and Cu@PET-*g*-PAA (**d**) composite TeMs prepared using e-beam and γ-rays, respectively (temperature—30 °C; MB concentration—3 mg/L; size of the catalyst membrane—1 × 1 cm).

The UV–Vis absorption spectrum of MB reveals an intense absorption peak corresponding to a single MB molecule at around 664 nm (Figure 8a,b). At about 612 nm, a shoulder attributed to MB dimer accompanies this intense peak. Two additional bands appear in the ultraviolet region at around 292 and 245 nm (associated with substituted benzene rings) [20,57]. These absorption peaks gradually decrease as the photodegradation reaction progresses.

Using the maximum absorbance at 664 nm in Formula (3), the calculated variation in dye degradation efficiency with respect to the reaction time demonstrated that the highest efficiency of Cu(OH)_2_@PET-*g*-PAA was observed for the membrane prepared at 250 kGy (Figure 8c). At 180 min, more than 91.9% of the initial dye degraded under UV light, while only 83.9% of MB was removed from the reaction mixture when using Cu@PET-*g*-PAA membranes (Figure 8d), although the radiation dose and treatment time were the same.

In general, the observed photocatalytic activity of a photocatalyst is influenced by many factors, such as surface area, phase structure, crystallinity, density of active groups, and surface charge. Pre-adsorption is indispensable for dyes to be photodegraded on the photocatalyst surface, since rapid sorption of dyes facilitates electron injection [58]. The results showed that about 8.3% of MB molecules were absorbed by Cu@PET-*g*-PAA composite and about 16.4% by of Cu(OH)_2_@PET-*g*-PAA prepared at 100 kGy.

As can be seen from the presented data, composite membranes obtained using electron beam demonstrate a higher photocatalytic activity at 30 °C compared to those containing copper nanoclusters obtained under γ-rays.

The degradation reaction of MB followed the Langmuir–Hinshelwood mechanism [59] with a pseudo-first-order kinetics model, which allows the apparent rate constant to be calculated from the change in dose rate:

(4) $$\ln\left(\frac{C_{0}}{C}\right)=k_{a}t$$

where *C*_0_ is the initial dye concentration (mg/L), *C* is the concentration of dye at time *t*, *t* is the irradiation time (min), and $k_{a}$ is the reaction rate constant (min^−1^).

**Figure 9:**
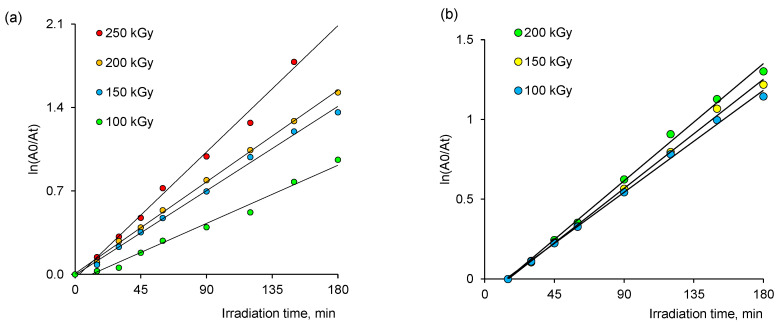
Variation of ln(C_0_/C) as a function of UV light irradiation time for Cu(OH)_2_@PET-*g*-PAA (**a**) and Cu@PET-*g*-PAA (**b**) membranes.

**Figure 10:**
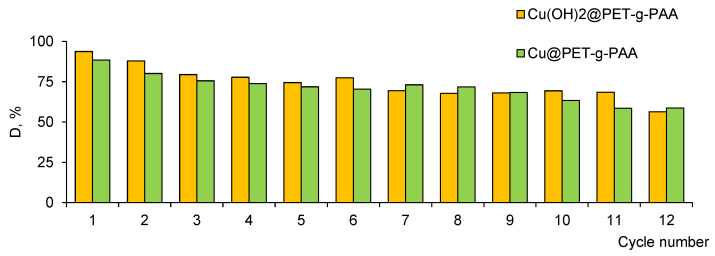
Reusability of studied catalysts for the degradation of MB.

The rate constant $k_{a}$ was determined as the slope of the graphs in Figure 9 by regression analysis. As can be seen from the graphs, the degradation of MB followed pseudo-first-order kinetics, as confirmed by the linearity of the logarithm of normalized concentration ln(C_0_/C) against t plots. The apparent rate constants calculated from these curves are presented in Table 3. It is clearly seen in Figure 10a,b that the degradation rate increases with increasing radiation dose absorbed during the synthesis of NCs. This can be attributed to the increased amount of loaded catalyst as seen from the data presented in Figure 7.

#### 3.3.1. Thermodynamic Parameters of Photocatalytic Degradation

The effect of temperature on the decomposition of MB in the presence of composite catalysts was explored in the temperature range of 20–40 °C.

The variation of the apparent rate constant k_a_ and the maximum degradation efficiency values (D, %) at different irradiation doses (100, 150, 200, 250 kGy) and temperatures (20, 30, 40 °C) were presented in Table 3. The apparent rate constant k_a_ increased for all types of composites as the temperature increased from 20 to 40 °C. Activation energy Ea changed between 8.41 and 12.03 kJ/mol depending on the type (e-beam or γ-rays) and dose (100–250 kGy) of irradiation, as can be seen in Table 4. The data obtained were consistent with the results of previous studies, where Ea was reported as 20.6 and 26.95 kJ/mol for Ag@PET TeMs of 2 × 2 cm size [21] and La/Cu Co-doped attapulgite composites [60], respectively. As an advantage, the results in our study presented a lower activation energy. From the presented data, it can be said that there is a common increasing tendency in degradation with increasing temperature for all catalyst systems.

The Eyring equation was used to calculate activation enthalpy (∆*H*^≠^; kJ/mol) and entropy (∆*S*^≠^; J mol/K) from the slope and intercept of *ln*(*ka/T*) versus 1/*T* plot, respectively, as expressed in the following Equation (5):

(5) $$\ln\left(\frac{k_{a}}{T}\right)=\ln\left(\frac{k_{B}}{h}\right)+\left(\frac{{∆S}^{\neq }}{R}\right)-(\frac{{∆H}^{\neq }}{RT})$$

where *k_B_* and *h* are the Boltzmann and Planck constants, respectively.

The enthalpy change calculated for all degradations was endothermic and ranged from 5.90 to 9.51 kJ/mol (Table 4). The entropy change was calculated as −0.26 kJ/molK for all degradations. Given the positive ∆*H^≠^* and negative ∆S^≠^ values, it was revealed that during the degradation of MB, endothermic interactions and a decrease in entropy occur at the solid–liquid interface. In all degradation reactions, Gibbs free energy change (∆G) was positive, indicating that the degradation of MB is a nonspontaneous process.

Rapid separation and efficient recycling of catalysts after a catalytic reaction are considered important requirements as well as the high catalytic performances. In this context, heterogeneous catalysts are more desirable because they have the advantage of easy recovery, although they are generally less efficient than the homogeneous type [61]. Flexible and mechanical stable TeMs offer promising opportunities for simplified separation and potential reuse of catalysts. In this study, the performance stability of composite membranes was evaluated by multi-cycle experiments.

As can be seen form the Figure 10, both types of catalysts remain highly active, even after being recycled 12 times without any additional activation treatment, and the value of the degradation efficiency was reduced by only 38.5% and 33.6% for Cu(OH)_2_@PET-*g*-PAA and Cu@PET-*g*-PAA membranes, respectively.

#### 3.3.2. Mechanism of MB Decomposition in the Presence of Composite TeMs

**Figure 11:**
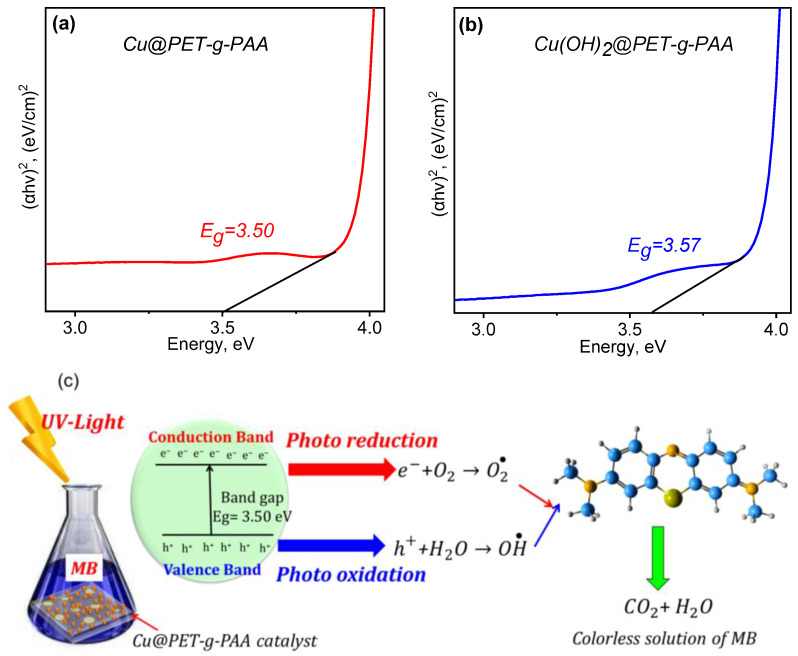
Tauc plot of optical transmittance spectra of Cu@PET-*g*-PAA (**a**) and Cu(OH)_2_@PET-*g*-PAA (**b**) and schematic representation of MB dye degradation mechanism using Cu@PET-*g*-PAA composite TeMs (**c**).

The optical band gap (E_g_) of the studied composite membranes was estimated using UV–Vis spectroscopy. The E_g_ of semiconductor materials should be laid between 1.6 and 2.5 eV to absorb light in the visible region of the solar spectrum. Such materials are defined as narrow band gap materials, while semiconductor materials with wide band gap (~3.2 eV) can only absorb light in the ultraviolet region [62,63]. Accordingly, in order to increase the conversion of sunlight energy with high efficiency, the absorption range must be extended to include regions from visible to infrared [63]. UV–Vis diffuse reflection spectra and corresponding Eg values of Cu(OH)_2_@PET-*g*-PAA and Cu@PET-*g*-PAA membranes prepared at 100 kGy are presented in Figure 11a,b. The band gap energy values were found by subtracting the linear part of (αhν)^2^ versus energy (hν). The calculated band gap energy of copper nanoclusters in Cu@PET-*g*-PAA was calculated as 3.50 eV, similar to that reported in the literature [64]. The Eg value for Cu(OH)_2_-based samples was found to be 3.57 eV. In previous studies, the band gap was reported as 3.3 eV for Cu(OH)_2_ nanowires [65] and 3.08 eV for Cu(OH)_2_ thin films [66]. The difference in Eg values can be attributed to the specific form, size, and synthesis methodology of copper-based nanosctructures. The probable charge carrier transfer mechanism for the degradation of MB using Cu@PET-*g*-PAA composite membrane is demonstrated shown in Figure 11c. As previously suggested [67], Cu nanoclusters supported on PET-*g*-PAA membrane absorb the photon energy, which is equal to its energy band gap and generates electrons and holes, thereby promoting the transfer of electrons from the valence band to the conduction band, leaving holes in the valence band. These electrons and holes in the conduction band and valence band react with the available acceptor and donor species absorbed on the surface of the photocatalyst to produce superoxide and hydroxyl radicals [67,68]. Since Cu@PET-*g*-PAA is a porous composite with a high surface area, it provides more active sites for electrons and holes to react with acceptor and donor species and helps to form more superoxide and hydroxyl radicals [69]. Then, the formed radicals, which are highly reactive in nature, react with the dye and degrade the dye into non-toxic compounds [20].

Results of recent studies on the photocatalytic degradation of MB using copper-based nanocatalysts are summarized in Table 5. As can be seen, the MB degradation efficiency reported by this study is one of the highest, and the amount of loaded catalysts is the smallest compared to other types. However, a direct comparison with other studies cannot be made as the experimental conditions, such as reaction time, MB concentration, testing temperature, catalyst loadings, are not the same.

## 4. Conclusions

In this study, radiation-induced synthesis of hybrid composite membranes decorated with Cu or Cu(OH)_2_ nanoclusters was first started by functionalization of the surface and nanochannel interiors of PET TeMs via effectual oxidation followed by graft copolymerization of acrylic acid. The grafted PAA contributed both to the sorption of Cu(II) ions and to the stabilization of the nanoparticles obtained by irradiation afterwards. Optimal conditions for radiation-induced grafting of PAA from PET TeMs by accelerated electrons were determined to achieve a sufficiently high degree of PAA grafting while maintaining the mechanical strength of the polymer template (CuSO_4_ concentration not more than 1%, AA concentration not exceeding 10%, radiation dose between 100–117 kGy). Irradiation of Cu(II) ion-absorbed PET-*g*-PAA membranes with accelerated electrons allowed for the synthesis of homogeneous copper hydroxide nanoparticles with small and uniform sizes throughout the entire volume of the template at a dose of 100 kGy. At higher doses, the size of the nanoparticles increased to 100 nm. In contrast, gamma-ray irradiation produced composites with crystalline copper nanoclusters, but their sizes were not uniform. The most homogeneous nanoparticles were synthesized at a dose of 150 kGy in case of gamma-irradiation. Based on the results of XRD and SEM analysis, it was confirmed that Cu or Cu(OH)_2_ nanoparticles were loaded onto the inner surface walls of the nanochannels, along with the surface of PET TeMs.

Synthesized Cu(OH)_2_@PET-*g*-PAA membranes exhibited crucial photocatalytic activity in the degradation of MB under UV light. In the presence of the hybrid Cu(OH)_2_@PET-*g*-PAA composite TeMs, the dye degradation reached 91.9% under UV light, while Cu@PET-*g*-PAA samples yielded only 83.9% MB degradation. Overall, our results showed that the synthesized composite membranes showed significant catalytic activity and were easy to use, making them a promising option for the treatment of wastewater-contaminated with organic dyes.

## Figures and Tables

**Table 1:** X-ray diffraction data of hybrid membranes after irradiation with accelerated electrons.

Dose, kGy	Phase/Phase Content, %	(hkl)	2θ° ^a^	d, Å ^b^	L, nm ^c^	FWHM ^d^	а, Å ^e^	Crystallinity Degree, %
100	Cu(OH)_2_ 100%	022	38.20	2.354	51.48	0.181	a = 2.950, b = 10.439, c = 5.283	67.4
		131	44.38	2.040	30.88	0.309		
		152	64.57	1.442	35.59	0.293		
		153	77.50	1.230	46.44	0.244		
		044	81.55	1.179	80.10	0.146		
150	Cu(OH)_2_ 100%	022	38.20	2.354	42.84	0.218	a = 2.947, b = 10.582, c = 5.252	59.3
		131	44.38	2.040	22.80	0.418		
		152	64.64	1.441	35.88	0.291		
		222	75.23	1.262	71.62	0.155		
		153	77.64	1.229	31.02	0.365		
200	Cu(OH)_2_ 100%	022	38.23	2.350	25.13	0.372	a = 2.924, b = 10.486, c = 5.233	43.2
		131	43.39	2.084	44.91	0.212		
250	Cu(OH)_2_ 100%	111	36.42	2.451	25.37	0.336	a = 2.917, b = 10.530, c = 5.211	44.6
		022	38.20	2.354	27.70	0.337		
		131	43.46	2.081	45.90	0.207		

^a^ Miller indices for corresponding planes; ^b^ spacing between planes; ^c^ average crystallite size; ^d^ full-width at half-maximum; ^e^ crystal lattice parameter.

**Table 2:** X-ray diffraction data of hybrid membranes after irradiation by γ-rays.

Dose, kGy	Phase/ Phase Content, %	(hkl) ^a^	2θ°	d, Å ^b^	L, nm ^c^	FWHM ^d^	а, Å ^e^	Crystallinity Degree, %
100	Cu 100%	111	43.38	2.084	40.46	0.235	a = 3.613	65.6
		200	50.56	1.804	27.46	0.356		
		220	74.16	1.278	30.51	0.363		
		311	90.01	1.089	26.28	0.475		
150	Cu 100%	111	43.38	2.084	95.38	0.100	a = 3.603	81.3
		200	50.54	1.804	44.54	0.219		
200	Cu 100%	111	43.46	2.081	64.45	0.147	a = 3.598	75.3
		200	50.56	1.804	26.90	0.363		
		220	74.16	1.278	57.90	0.191		
		311	89.88	1.091	49.46	0.252		

^a^ Miller indices for corresponding planes; ^b^ spacing between planes; ^c^ average crystallite size; ^d^ full-width at half-maximum; ^e^ crystal lattice parameter.

**Table 3:** Kinetic parameters of the degradation of MB at different temperatures.

Dose, kGy	Testing Temperature, °C											
	γ-rays/ Cu@PET-*g*-PAA						e-Beam/ Cu(OH)_2_@PET-*g*-PAA					
	D,%			k_a_ × 10^−2^ (min^−1^)			D,%			k_a_ × 10^−2^ (min^−1^)		
	20	30	40	20	30	40	20	30	40	20	30	40
100	74.4	81.4	82.2	0.71	0.87	0.95	72.9	72.5	77.2	0.54	0.65	0.74
150	75.1	80.7	98.3	0.76	0.90	1.00	74.7	80.6	89.5	0.79	0.88	1.04
200	81.2	83.9	88.4	0.83	0.96	1.08	79.2	87.9	85.5	0.85	1.00	1.07
250	-	-	-	-	-	-	89.3	91.9	93.8	1.18	1.38	1.47

**Table 4:** Thermodynamic parameters of the degradation reaction of MB.

Dose, kGy	γ-rays/ Cu@PET-*g*-PAA			e-Beam/ Cu(OH)_2_@PET-*g*-PAA			
	100	150	200	100	150	200	250
E_A_, kJ/mol	11.15	10.49	10.05	12.03	10.45	8.81	8.41
∆H, kJ/mol	8.63	7.97	7.53	9.51	7.94	6.29	5.90
∆S, kJ/mol·K	−0.26	−0.26	−0.26	−0.26	−0.26	−0.26	−0.26
∆G, kJ/mol (293K)	83.73	83.58	83.38	84.42	83.54	83.30	82.50

**Table 5:** Recent research on photocatalytic MB degradation using catalysts containing copper nanostructures.

Catalyst	Catalyst Test Conditions				Catalyst Efficiency			Ref.
	MB, mg/L	Contact Time, min	Catalyst Dosage	Light Source	Lifetime, Cycle	D,%	k × 10^−4^, min^−1^	
Copper-doped TiO_2_ powder	10.0	300	300 mg	Visible light	3	25.1	14.0	[70]
Copper-impregnated TiO_2_ powder						42.4	3.7	
CuO/MgO/PVC	0.05	150	5 × 2.5 cm^2^	UV light	-	44.0	52.0	[71]
Cu_2_O-BiVO_4_	5.0	240	4.5 mg	Visible light	3	97.0	9.0	[72]
CuO NPs (biogenic)	1.0	100	100.0 mg	Visible light	5	69.3	140.0	[73]
CuO/Zn film	5.0	240	30 × 25 mm^2^	Visible light	5	82.0	67.5	[74]
Cu_2_O	1.0	120	0.5 mg	Sunlight	4	70.0	108.0	[75]
CuO					4	63.0	83.0	
CuO/CeO	1.0	150	1.0 g/L	Visible light	-	85.66	-	[76]
Cu nanosheets	42.0	100	500.0 mg	UV light	-	40.0	-	[67]
CuO/Bi_2_O_3_	20.0	120	0.2 g/L	UV light	4	88.32	173.0	[77]
CuO/PET nanocomposite	10.0	30	40.0 mg	UV light	-	99.0	2380.0	[78]
CuO-3La	0.625	90	13 mg/L	Visible light	5	99.0	182.0	[79]
Cu(OH)_2_@PET-g-AA (e-beam, 250 kGy)	3.0	180	0.5 mg	UV light	12	91.85	138.0	This study
Cu@PET-g-AA (γ-rays, 200 kGy)			0.4 mg		12	83.96	96.0	

## Data Availability

Not applicable.

## Supplementary Materials

The following supporting information can be downloaded at: https://www.mdpi.com/article/10.3390/membranes13070659/s1, Figure S1. Calibration curve of e-beam (B3WinDose dosimeters); Figure S2. Membranes tensile testing machine; Figure S3. Laboratory setup for photocatalytic experiments; Figure S4. Typical XRD patterns of the Cu(OH)2@PET-g-PAA (a) and Cu@PET-g-PAA (b) membranes (irradiation dose—100 kGy).

## Abbreviations

TeM	Track-etched membrane
AA	Acrylic acid
PAA	Poly(acrylic acid)
PET	Poly(ethylene terephthalate)
XRD	X-ray diffraction
PET-*g*-PAA	PAA-grafted PET TeM
Cu(OH)_2_@PET-*g*-PAA	PAA-grafted composite membrane with loaded Cu(OH)_2_ nanoclusters synthesized using e-beam
Cu@PET-*g*-PAA	PAA-grafted composite membrane with loaded Cu nanoclusters synthesized using γ-rays
NC	Nanocluster
SEM	Scanning electron microscopy
DC	Degree of crystallinity (%)
L	Average crystallite size (nm)
k_a_	Aate constant (min^−1^)
D	Degradation rate (%)
E_A_	Activation energy (kJ/mol)
∆H	Activation enthalpy (kJ/mol)
∆S	Activation entropy (kJ/mol·K)
∆G	Gibbs free energy (kJ/mol)

## References

[1] Abadulla E., Tzanov T., Costa S., Cavaco A., Gübitz G. (2000). Decolorization and Detoxification of Textile Dyes with a Laccase from Trametes Hirsuta Decolorization and Detoxification of Textile Dyes with a Laccase from *Trametes hirsuta*. Appl. Environ. Microbiol..

[2] Forgacs E., Cserháti T., Oros G. (2004). Removal of Synthetic Dyes from Wastewaters: A Review. Environ. Int..

[3] Kant R. (2012). Textile Dyeing Industry an Environmental Hazard. Nat. Sci..

[4] Lazar M., Varghese S., Nair S. (2012). Photocatalytic Water Treatment by Titanium Dioxide: Recent Updates. Catalysts.

[5] Katheresan V., Kansedo J., Lau S.Y. (2018). Efficiency of Various Recent Wastewater Dye Removal Methods: A Review. J. Environ. Chem. Eng..

[6] Zhou Y., Lu J., Zhou Y., Liu Y. (2019). Recent Advances for Dyes Removal Using Novel Adsorbents: A Review. Environ. Pollut..

[7] Piaskowski K., Świderska-Dąbrowska R., Zarzycki P.K. (2018). Dye Removal from Water and Wastewater Using Various Physical, Chemical, and Biological Processes. J. AOAC Int..

[8] Bhatia D., Sharma N.R., Singh J., Kanwar R.S. (2017). Biological Methods for Textile Dye Removal from Wastewater: A Review. Crit. Rev. Environ. Sci. Technol..

[9] Gayathiri E., Prakash P., Selvam K., Awasthi M.K., Gobinath R., Karri R.R., Ragunathan M.G., Jayanthi J., Mani V., Poudineh M.A. (2022). Plant Microbe Based Remediation Approaches in Dye Removal: A Review. Bioengineered.

[10] Deng Y., Zhao R. (2015). Advanced Oxidation Processes (AOPs) in Wastewater Treatment. Curr. Pollut. Rep..

[11] Atalay S., Ersöz G. (2015). Advanced Oxidation Processes for Removal of Dyes from Aqueous Media. Green Chemistry for Dyes Removal from Wastewater.

[12] Ledakowicz S., Paździor K. (2021). Recent Achievements in Dyes Removal Focused on Advanced Oxidation Processes Integrated with Biological Methods. Molecules.

[13] Panda J., Sahu S.N., Tripathy R.R., Sahoo T., Sahu J.R., Pattanayak S.K., Sahu R. (2021). Metal–Organic Frameworks for Heterogeneous Photocatalysis of Organic Dyes. Photocatalytic Degradation of Dyes.

[14] Beydaghdari M., Hooriabad Saboor F., Babapoor A., Karve V., Asgari M. (2022). Recent Advances in MOF-Based Adsorbents for Dye Removal from the Aquatic Environment. Energies.

[15] Rashid R., Shafiq I., Akhter P., Iqbal M.J., Hussain M. (2021). A State-of-the-Art Review on Wastewater Treatment Techniques: The Effectiveness of Adsorption Method. Environ. Sci. Pollut. Res..

[16] Grzegorzek M., Wartalska K., Kaźmierczak B. (2023). Review of Water Treatment Methods with a Focus on Energy Consumption. Int. Commun. Heat Mass Transf..

[17] Gupta V.K. (2009). Application of Low-Cost Adsorbents for Dye Removal—A Review. J. Environ. Manag..

[18] Zhang Y., Wang Y., Guo C., Wang Y. (2022). Molybdenum Carbide-Based Photocatalysts: Synthesis, Functionalization, and Applications. Langmuir.

[19] Byrne C., Subramanian G., Pillai S.C. (2018). Recent Advances in Photocatalysis for Environmental Applications. J. Environ. Chem. Eng..

[20] Khan I., Saeed K., Zekker I., Zhang B., Hendi A.H., Ahmad A., Ahmad S., Zada N., Ahmad H., Shah L.A. (2022). Review on Methylene Blue: Its Properties, Uses, Toxicity and Photodegradation. Water.

[21] Mashentseva A.A., Barsbay M., Aimanova N.A., Zdorovets M.V. (2021). Application of Silver-Loaded Composite Track-Etched Membranes for Photocatalytic Decomposition of Methylene Blue under Visible Light. Membranes.

[22] Al-Tohamy R., Ali S.S., Li F., Okasha K.M., Mahmoud Y.A.-G., Elsamahy T., Jiao H., Fu Y., Sun J. (2022). A Critical Review on the Treatment of Dye-Containing Wastewater: Ecotoxicological and Health Concerns of Textile Dyes and Possible Remediation Approaches for Environmental Safety. Ecotoxicol. Environ. Saf..

[23] Zhang S., Malik S., Ali N., Khan A., Bilal M., Rasool K. (2022). Covalent and Non-Covalent Functionalized Nanomaterials for Environmental Restoration. Top. Curr. Chem..

[24] Korolkov I.V., Güven O., Mashentseva A.A., Atıcı A.B., Gorin Y.G., Zdorovets M.V., Taltenov A.A. (2017). Radiation Induced Deposition of Copper Nanoparticles inside the Nanochannels of Poly(Acrylic Acid)-Grafted Poly(Ethylene Terephthalate) Track-Etched Membranes. Radiat. Phys. Chem..

[25] Khdary N.H., Almuarqab B.T., El Enany G. (2023). Nanoparticle-Embedded Polymers and Their Applications: A Review. Membranes.

[26] Obotey Ezugbe E., Rathilal S. (2020). Membrane Technologies in Wastewater Treatment: A Review. Membranes.

[27] Mashentseva A.A., Zdorovets M. (2019). V Catalytic Activity of Composite Track-Etched Membranes Based on Copper Nanotubes in Flow and Static Modes. Pet. Chem..

[28] Borgekov D., Mashentseva A., Kislitsin S., Kozlovskiy A., Russakova A., Zdorovets M. (2015). Temperature Dependent Catalytic Activity of Ag/PET Ion-Track Membranes Composites. Acta Phys. Pol. A.

[29] Bräuer P., Muench F., Stojkovikj S., Gupta S., Mayer M.T., Ensinger W., Roth C., El-Nagar G.A. (2022). Shape-Controlled Electroless Plating of Hetero-Nanostructures: AgCu- and AgNi-Decorated Ag Nanoplates on Carbon Fibers as Catalysts for the Oxygen Evolution Reaction. ACS Appl. Nano Mater..

[30] Muench F. (2021). Electroless Plating of Metal Nanomaterials. ChemElectroChem.

[31] Boettcher T., Schaefer S., Antoni M., Stohr T., Kunz U., Dürrschnabel M., Molina-Luna L., Ensinger W., Muench F. (2019). Shape-Selective Electroless Plating within Expanding Template Pores: Etching-Assisted Deposition of Spiky Nickel Nanotube Networks. Langmuir.

[32] Pérez-Mitta G., Toimil-Molares M.E., Trautmann C., Marmisollé W.A., Azzaroni O. (2019). Molecular Design of Solid-State Nanopores: Fundamental Concepts and Applications. Adv. Mater..

[33] Rabajczyk A., Zielecka M., Cygańczuk K., Pastuszka Ł., Jurecki L. (2021). Nanometals-Containing Polymeric Membranes for Purification Processes. Materials.

[34] Zahid M., Rashid A., Akram S., Rehan Z.A., Razzaq W. (2018). A Comprehensive Review on Polymeric Nano-Composite Membranes for Water Treatment. J. Membr. Sci. Technol..

[35] Parmanbek N., Sütekin D.S., Barsbay M., Mashentseva A.A., Zheltov D.A., Aimanova N.A., Jakupova Z.Y., Zdorovets M.V. (2022). Hybrid PET Track-Etched Membranes Grafted by Well-Defined Poly(2-(dimethylamino)ethyl methacrylate) Brushes and Loaded with Silver Nanoparticles for the Removal of As(III). Polymers.

[36] Güven O. (2021). Radiation-Assisted Synthesis of Polymer-Based Nanomaterials. Appl. Sci..

[37] Feldman V.I., Zezin A.A., Abramchuk S.S., Zezina E.A. (2013). X-ray Induced Formation of Metal Nanoparticles from Interpolyelectrolyte Complexes with Copper and Silver Ions: The Radiation-Chemical Contrast. J. Phys. Chem. C.

[38] Zezin A.A. (2016). Synthesis of Hybrid Materials in Polyelectrolyte Matrixes: Control over Sizes and Spatial Organization of Metallic Nanostructures. Polym. Sci. Ser. C.

[39] Bakar A., Güven O., Zezin A.A., Feldman V.I. (2014). Controlling the Size and Distribution of Copper Nanoparticles in Double and Triple Polymer Metal Complexes by X-ray Irradiation. Radiat. Phys. Chem..

[40] Korolkov I.V., Mashentseva A.A., Güven O., Gorin Y.G., Kozlovskiy A.L., Zdorovets M.V., Zhidkov I.S., Cholach S.O. (2018). Electron/Gamma Radiation-Induced Synthesis and Catalytic Activity of Gold Nanoparticles Supported on Track-Etched Poly(Ethylene Terephthalate) Membranes. Mater. Chem. Phys..

[41] Abedini A., Daud A.R., Abdul Hamid M.A., Kamil Othman N., Saion E. (2013). A Review on Radiation-Induced Nucleation and Growth of Colloidal Metallic Nanoparticles. Nanoscale Res. Lett..

[42] Naghavi K., Saion E., Rezaee K., Yunus W.M.M. (2010). Influence of Dose on Particle Size of Colloidal Silver Nanoparticles Synthesized by Gamma Radiation. Radiat. Phys. Chem..

[43] Kirshanov K., Toms R., Aliev G., Naumova A., Melnikov P., Gervald A. (2022). Recent Developments and Perspectives of Recycled Poly(Ethylene Terephthalate)-Based Membranes: A Review. Membranes.

[44] Lu Y.Z., Wei W.T., Chen W. (2012). Copper Nanoclusters: Synthesis, Characterization and Properties. Chin. Sci. Bull..

[45] Russakova A.V., Altynbaeva L.S., Barsbay M., Zheltov D.A., Zdorovets M.V., Mashentseva A.A. (2021). Kinetic and Isotherm Study of As(III) Removal from Aqueous Solution by PET Track-Etched Membranes Loaded with Copper Microtubes. Membranes.

[46] Mashentseva A.A., Barsbay M., Zdorovets M.V., Zheltov D.A., Güven O. (2020). Cu/CuO Composite Track-Etched Membranes for Catalytic Decomposition of Nitrophenols and Removal of As(III). Nanomaterials.

[47] Barsbay M., Çaylan Özgür T., Sütekin S.D., Güven O. (2020). Effect of Brush Length of Stabilizing Grafted Matrix on Size and Catalytic Activity of Metal Nanoparticles. Eur. Polym. J..

[48] Mantilaka M.M.M.G.P.G., Rajapakse R.M.G., Karunaratne D.G.G.P., Pitawala H.M.T.G.A. (2014). Preparation of Amorphous Calcium Carbonate Nanoparticles from Impure Dolomitic Marble with the Aid of Poly(Acrylic Acid) as a Stabilizer. Adv. Powder Technol..

[49] Korolkov I.V., Mashentseva A.A., Güven O., Niyazova D.T., Barsbay M., Zdorovets M.V. (2014). The Effect of Oxidizing Agents/Systems on the Properties of Track-Etched PET Membranes. Polym. Degrad. Stab..

[50] Kavaklı C., Barsbay M., Tilki S., Güven O., Kavaklı P.A. (2016). Activation of Polyethylene/Polypropylene Nonwoven Fabric by Radiation-Induced Grafting for the Removal of Cr(VI) from Aqueous Solutions. Water Air Soil Pollut..

[51] Barsbay M., Güven O. (2020). Nanostructuring of Polymers by Controlling of Ionizing Radiation-Induced Free Radical Polymerization, Copolymerization, Grafting and Crosslinking by RAFT Mechanism. Radiat. Phys. Chem..

[52] Wittenberg N.F.G., Preusser C., Kattner H., Stach M., Lacík I., Hutchinson R.A., Buback M. (2016). Modeling Acrylic Acid Radical Polymerization in Aqueous Solution. Macromol. React. Eng..

[53] Korolkov I.V., Mashentseva A.A., Güven O., Taltenov A.A. (2015). UV-Induced Graft Polymerization of Acrylic Acid in the Sub-Micronchannels of Oxidized PET Track-Etched Membrane. Nucl. Instrum. Methods Phys. Res. Sect. B Beam Interact. Mater. Atoms.

[54] Korolkov I.V., Borgekov D.B., Mashentseva A.A., Güven O., Atlcl A.B., Kozlovskiy A.L., Zdorovets M.V. (2017). The Effect of Oxidation Pretreatment of Polymer Template on the Formation and Catalytic Activity of Au/PET Membrane Composites. Chem. Pap..

[55] Oladoye P.O., Ajiboye T.O., Omotola E.O., Oyewola O.J. (2022). Methylene Blue Dye: Toxicity and Potential Elimination Technology from Wastewater. Results Eng..

[56] Shakoor S., Nasar A. (2016). Removal of Methylene Blue Dye from Artificially Contaminated Water Using Citrus Limetta Peel Waste as a Very Low Cost Adsorbent. J. Taiwan Inst. Chem. Eng..

[57] Jiang J., Xie N., Jiang Y., Han J., Feng G., Shi Z., He C. (2022). Rapid Photodegradation of Methylene Blue by Laser-Induced Plasma. RSC Adv..

[58] Guo J., Yuan S., Jiang W., Yue H., Cui Z., Liang B. (2016). Adsorption and Photocatalytic Degradation Behaviors of Rhodamine Dyes on Surface-Fluorinated TiO_2_ under Visible Irradiation. RSC Adv..

[59] Wu C.-H., Chern J.-M. (2006). Kinetics of Photocatalytic Decomposition of Methylene Blue. Ind. Eng. Chem. Res..

[60] Shang J., Zhang W., Dong Z., Fan H.-J.S. (2023). Kinetics of Catalytic Oxidation of Methylene Blue with La/Cu Co-Doped in Attapulgite. Materials.

[61] Miceli M., Frontera P., Macario A., Malara A. (2021). Recovery/Reuse of Heterogeneous Supported Spent Catalysts. Catalysts.

[62] Parmanbek N., Sütekin S.D., Barsbay M., Aimanova N.A., Mashentseva A.A., Alimkhanova A.N., Zhumabayev A.M., Yanevich A., Almanov A.A., Zdorovets M.V. (2023). Environmentally Friendly Loading of Palladium Nanoparticles on Nanoporous PET Track-Etched Membranes Grafted by Poly(1-Vinyl-2-Pyrrolidone) via RAFT Polymerization for the Photocatalytic Degradation of Metronidazole. RSC Adv..

[63] Al-Mahamad L.L.G. (2022). Analytical Study to Determine the Optical Properties of Gold Nanoparticles in the Visible Solar Spectrum. Heliyon.

[64] Boltaev G.S., Ganeev R.A., Krishnendu P.S., Zhang K., Guo C. (2019). Nonlinear Optical Characterization of Copper Oxide Nanoellipsoids. Sci. Rep..

[65] Bhusari R., Thomann J.-S., Guillot J., Leturcq R. (2021). Morphology Control of Copper Hydroxide Based Nanostructures in Liquid Phase Synthesis. J. Cryst. Growth.

[66] Gurav K.V., Patil U.M., Shin S.W., Agawane G.L., Suryawanshi M.P., Pawar S.M., Patil P.S., Lokhande C.D., Kim J.H. (2013). Room Temperature Chemical Synthesis of Cu(OH)_2_ Thin Films for Supercapacitor Application. J. Alloys Compd..

[67] Chandan M.R., Kumar K.R., Shaik A.H. (2022). Two-Dimensional Cu Nanostructures for Efficient Photo-Catalytic Degradation of Methylene Blue. Environ. Sci. Adv..

[68] Wang Q., Tian S., Ning P. (2014). Degradation Mechanism of Methylene Blue in a Heterogeneous Fenton-like Reaction Catalyzed by Ferrocene. Ind. Eng. Chem. Res..

[69] Molinari R., Pirillo F., Falco M., Loddo V., Palmisano L. (2004). Photocatalytic Degradation of Dyes by Using a Membrane Reactor. Chem. Eng. Process. Process Intensif..

[70] Pava-Gómez B., Vargas-Ramírez X., Díaz-Uribe C. (2018). Physicochemical Study of Adsorption and Photodegradation Processes of Methylene Blue on Copper-Doped TiO_2_ Films. J. Photochem. Photobiol. A Chem..

[71] Rouabah N., Nazir R., Djaballah Y., Mir A.Q., Ameur I., Beldjebli O. (2023). Synthesis of a Thin Film of CuO/MgO/PVC Nanocomposites for Photocatalytic Applications. Iran. J. Catal..

[72] Razi R., Sheibani S. (2021). Photocatalytic Activity Enhancement by Composition Control of Mechano-Thermally Synthesized BiVO4-Cu_2_O Nanocomposite. Ceram. Int..

[73] Mashentseva A.A., Aimanova N.A., Temirgaziev B.S., Zhumazhanova A.T., Tuleuov B.I. (2020). Photocatalytic Activity of Copper(II) Oxide Nanoparticles Synthesized Using *Serratula coronata* L. Extract. Pet. Chem..

[74] Karazmoudeh N.J., Soltanieh M., Hasheminiasari M. (2023). Structural and Photocatalytic Properties of Undoped and Zn-Doped CuO Thin Films Deposited by Reactive Magnetron Sputtering. J. Alloys Compd..

[75] Akter J., Sapkota K.P., Hanif M.A., Islam M.A., Abbas H.G., Hahn J.R. (2021). Kinetically Controlled Selective Synthesis of Cu_2_O and CuO Nanoparticles toward Enhanced Degradation of Methylene Blue Using Ultraviolet and Sun Light. Mater. Sci. Semicond. Process..

[76] Raees A., Jamal M.A., Ahmed I., Silanpaa M., Algarni T.S. (2021). Synthesis and Characterization of Ceo_2_/Cuo Nanocomposites for Photocatalytic Degradation of Methylene Blue in Visible Light. Coatings.

[77] Poorsajadi F., Sayadi M.H., Hajiani M., Rezaei M.R. (2022). Synthesis of CuO/Bi_2_O_3_ Nanocomposite for Efficient and Recycling Photodegradation of Methylene Blue Dye. Int. J. Environ. Anal. Chem..

[78] Yasin S.A., Zeebaree S.Y.S., Zeebaree A.Y.S., Zebari O.I.H., Saeed I.A. (2021). The Efficient Removal of Methylene Blue Dye Using CuO/PET Nanocomposite in Aqueous Solutions. Catalysts.

[79] Rafique M., Khalid N.R., Irshad M., Shafiq F., Usman M., Fouad Y., Imran M., Assiri M.A., Ashraf W.M. (2023). Visible Light-Active Pure and Lanthanum-Doped Copper Oxide Nanostructures for Photocatalytic Degradation of Methylene Blue Dye and Hydrogen Production. Energy Sci. Eng..

