# Supplementary material for: e-Beam and γ-rays Induced Synthesis and Catalytic Properties of Copper Nanoclusters-Deposited Composite Track-Etched Membranes
Source: Membranes (Basel). 2023 Jul 11;13(7):659. doi: 10.3390/membranes13070659 (PMC10385425; doi:10.3390/membranes13070659)
Supplement: Supplementary file 1 [file membranes-13-00659-s001.zip › membranes-2442709-supplementary.pdf]

membranes

Supplementary Materials

e-Beam and γ-rays Induced Synthesis and Catalytic Properties of Copper Nanoclusters-Deposited Composite Track-Etched Membranes

Nursanat Parmanbek[1,2], Nurgulim A. Aimanova [1], Anastassiya A. Mashentseva [1,3,*], Murat Barsbay [4], Fatima U. Abuova [3,*], Dinara T. Nurpeisova [2], Zhanar Ye. Jakupova [2] and Maxim V. Zdorovets [1,5,6]

1 The Institute of Nuclear Physics of the Republic of Kazakhstan, 050032 Almaty, Kazakhstan
2 Department of Chemistry, L.N. Gumilyov Eurasian National University, 010008 Astana, Kazakhstan
3 Department of Nuclear physics, new materials and technologies, L.N. Gumilyov Eurasian National University, 010008 Astana, Kazakhstan
4 Department of Chemistry, Hacettepe University, 06800 Ankara, Turkey
5 Department of Intelligent Information Technologies, The Ural Federal University, 620002 Yekaterinburg, Russia
6 Engineering Profile Laboratory, L.N. Gumilyov Eurasian National University, 010008 Astana, Kazakhstan
* Correspondence: a.mashentseva@inp.kz (A.A.M.); abuova_fu@enu.kz (F.U.A.)

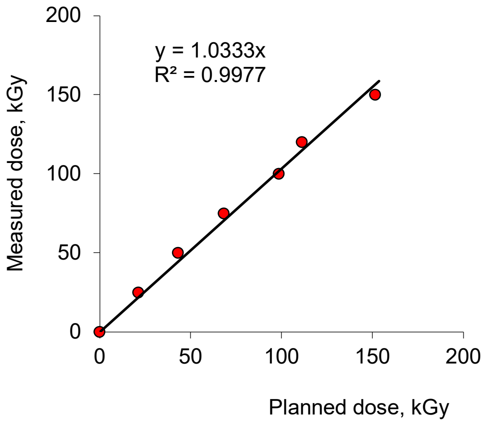

Figure S1. Calibration curve of e-beam (B3WinDose dosimeters).

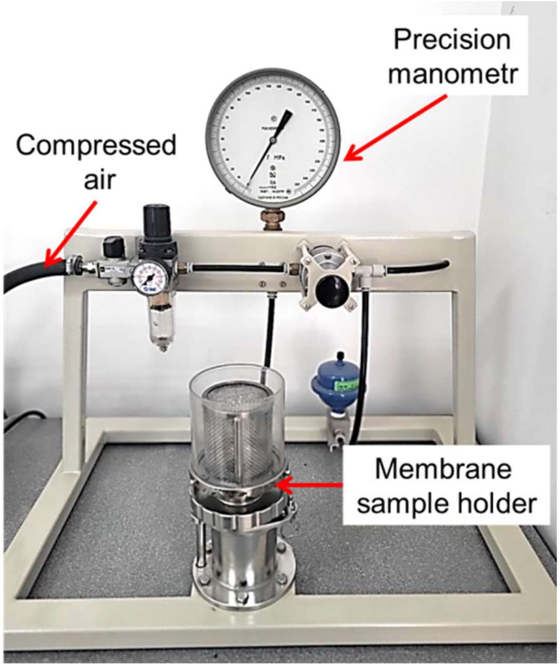

Figure S2. Membranes tensile testing machine.

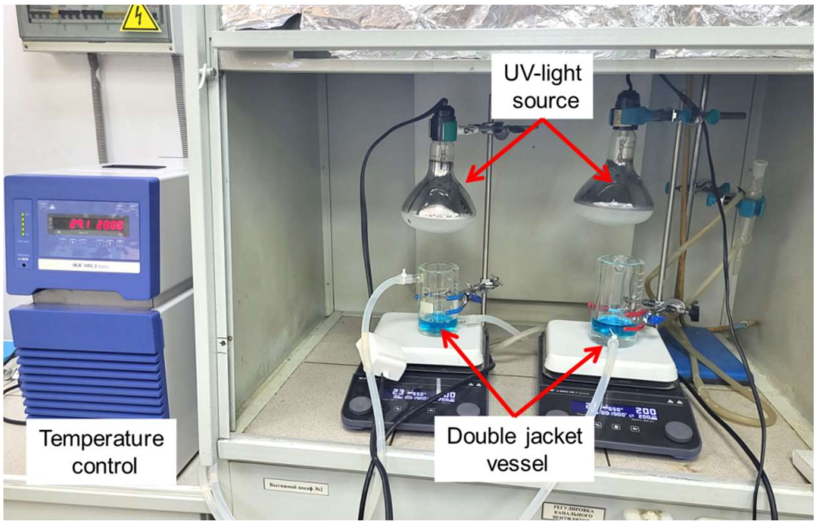

Figure S3. Laboratory setup for photocatalytic experiments.

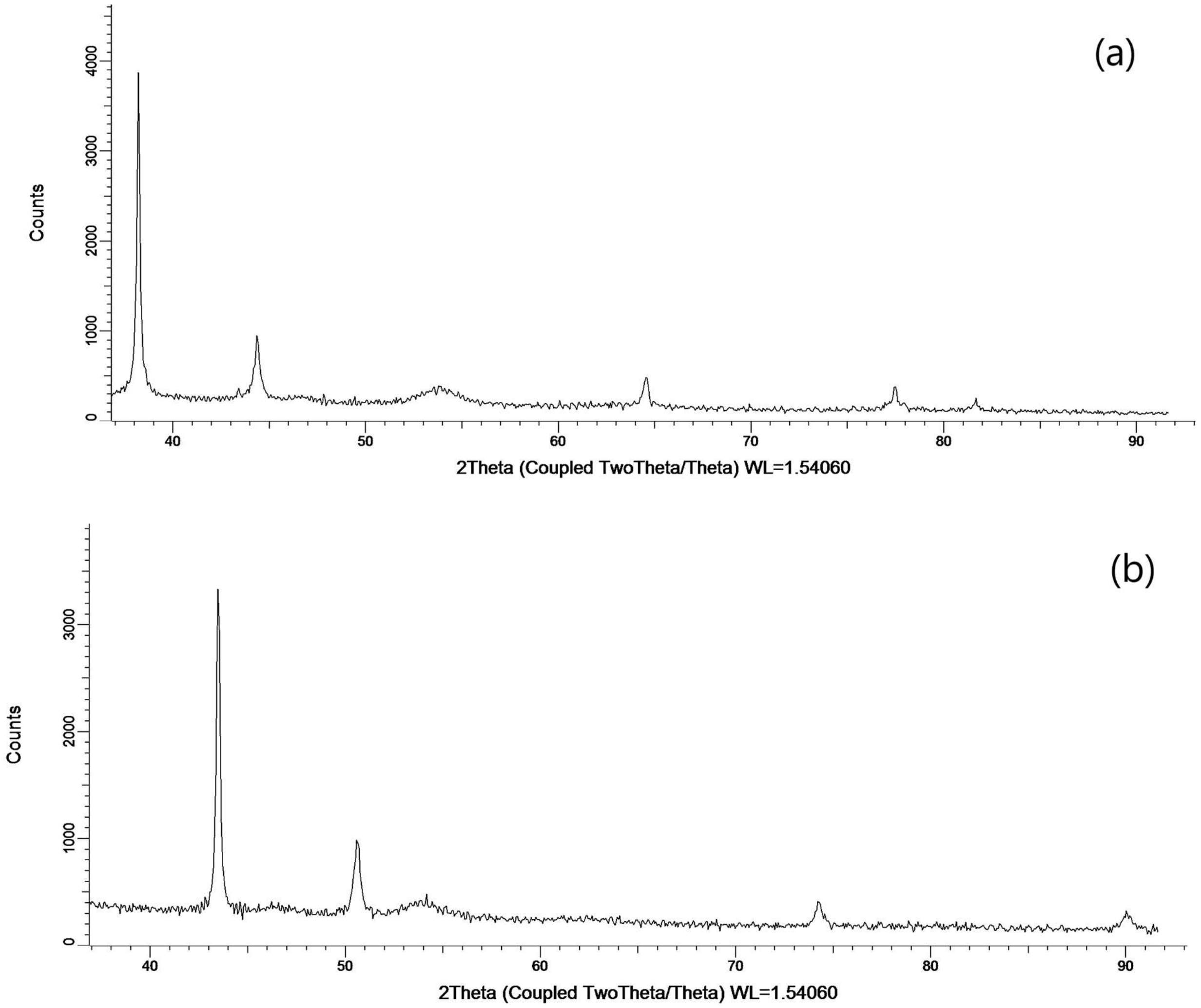

Figure S4. Typical XRD patterns of the $Cu(OH)_2$@PET-*g*-PAA (**a**) and Cu@PET-*g*-PAA (**b**) membranes (irradiation dose – 100 kGy).
